# Induced sputum KL-6 combined with HRCT scoring for diagnosing and monitoring idiopathic pulmonary fibrosis

**DOI:** 10.17305/bb.2025.12667

**Published:** 2025-09-12

**Authors:** Bingxin Zhang, Dejun Zhao, Danping Hu

**Affiliations:** 1 The Second Department of Respiration, The First People’s Hospital of Fuyang District, Hangzhou, China

**Keywords:** Diagnosis, high-resolution computed tomography score, idiopathic pulmonary fibrosis, induced sputum, Krebs von den Lungen-6

## Abstract

Idiopathic pulmonary fibrosis (IPF) is a progressive and fatal interstitial lung disease for which reliable early diagnostic biomarkers are still lacking. This study aimed to evaluate the diagnostic and monitoring value of induced sputum Krebs von den Lungen-6 (KL-6) levels in patients with IPF and to investigate their relationship with pulmonary function parameters and high-resolution computed tomography (HRCT) scoring. In this prospective observational study, 20 patients with IPF and 20 age-matched healthy subjects (HS) were enrolled between October 2021 and April 2023. Induced sputum samples were collected for KL-6 measurement using enzyme-linked immunosorbent assay, while all participants underwent pulmonary function testing and HRCT scoring. KL-6 levels were significantly higher in the IPF group compared with the HS group [776.29 (interquartile range, IQR: 681.98–858.57) vs 322.21 (IQR: 253.67–338.64) U/mL, *P* < 0.001]. In IPF patients, induced sputum KL-6 levels showed strong negative correlations with multiple lung function indices, including forced expiratory volume in one second (FEV1), forced vital capacity (FVC), and diffusing capacity for carbon monoxide (DL_CO_) (all *P* < 0.05), and a strong positive correlation with HRCT scores (*r* ═ 0.908, *P* < 0.001). Receiver operating characteristic (ROC) analysis demonstrated that combining KL-6 levels with HRCT scores yielded an area under the curve (AUC) of 0.936 (95% confidence interval, CI: 0.914–0.944), with specificity of 97.5% and sensitivity of 80.0%. In conclusion, induced sputum KL-6 levels reflect the degree of pulmonary fibrosis and are closely associated with functional and imaging indicators in IPF. The combination of KL-6 with HRCT scoring enhances diagnostic accuracy, underscoring its potential clinical utility as a noninvasive biomarker for early detection and monitoring of IPF.

## Introduction

Idiopathic pulmonary fibrosis (IPF) is a progressive and irreversible interstitial lung disease (ILD) characterized by damage to the alveolar structure, abnormal fibrous tissue deposition, and persistent inflammatory responses [1]. As the disease advances, the architecture of lung tissue is altered, resulting in impaired gas exchange and a gradual decline in pulmonary function, which may ultimately lead to respiratory failure or death [2]. Clinically, patients typically present with progressive dyspnea, dry cough, deteriorating pulmonary function, and reticular or honeycomb-like imaging abnormalities, predominantly observed in the lower lung zones [3, 4]. High-resolution computed tomography (HRCT) is integral to the diagnosis of IPF, commonly revealing basal-predominant honeycombing and interstitial abnormalities [5–7].

IPF affects approximately 3 million individuals globally, with incidence rates significantly increasing with age [8]. Presently, treatment strategies, aside from lung transplantation, primarily aim to slow disease progression and prevent respiratory failure [9]. The median survival following diagnosis is only 3–5 years [10], and the natural progression of the disease is highly variable and unpredictable. Consequently, early diagnosis and intervention are crucial for enhancing patient outcomes. However, the absence of reliable early diagnostic biomarkers poses a significant challenge to the effective management and treatment of IPF. Therefore, the identification of novel, more sensitive, and specific biomarkers is vital for improving early detection rates.

Krebs von den Lungen-6 (KL-6), a glycoprotein encoded by the *MUC1* gene, is primarily expressed on type II alveolar epithelial cells [11, 12]. Research indicates that serum and bronchoalveolar lavage fluid (BALF) KL-6 levels are significantly elevated in patients with IPF and negatively correlate with pulmonary function parameters, including forced vital capacity (FVC) and diffusing capacity of the lungs for carbon monoxide (DL_CO_) [13, 14]. Furthermore, KL-6 levels in sputum demonstrate a strong correlation with the extent of pulmonary function impairment, highlighting its potential clinical relevance [15]. However, serum and natural sputum have limitations in accurately reflecting lung lesions, particularly in the lower respiratory tract. Induced sputum, collected through saline nebulization stimulation, may more effectively represent the local pathological conditions of the lower respiratory tract, providing more direct and sensitive biological information. Consequently, investigating the role of induced sputum KL-6 in the diagnosis and monitoring of IPF may yield a more accurate biomarker for clinical application. Based on this premise, the present study aims to evaluate the clinical utility of induced sputum KL-6 as a biomarker for IPF diagnosis by examining its associations with pulmonary function indices and HRCT imaging scores in IPF patients.

## Materials and methods

### Study population

This study was designed as a prospective observational analysis. Between October 2021 and April 2023, we consecutively recruited eligible participants from our institution, enrolling a total of 40 subjects, including 20 patients with IPF and 20 healthy controls. The inclusion criteria for the IPF group were as follows: patients aged 20 years or older; diagnosis of IPF based on the 2018 American Thoracic Society/European Respiratory Society (ATS/ERS) criteria [7] and the diagnostic and treatment guidelines established by the Respiratory Diseases Section of the Chinese Medical Association [16]; presence of key CT manifestations such as ground-glass opacities, honeycomb patterns, reticular patterns, and solid nodules; patients who were newly diagnosed with IPF and had not received systemic anti-fibrotic treatment; and patients who provided informed consent, were mentally competent, and exhibited normal consciousness.

**Figure 1.**
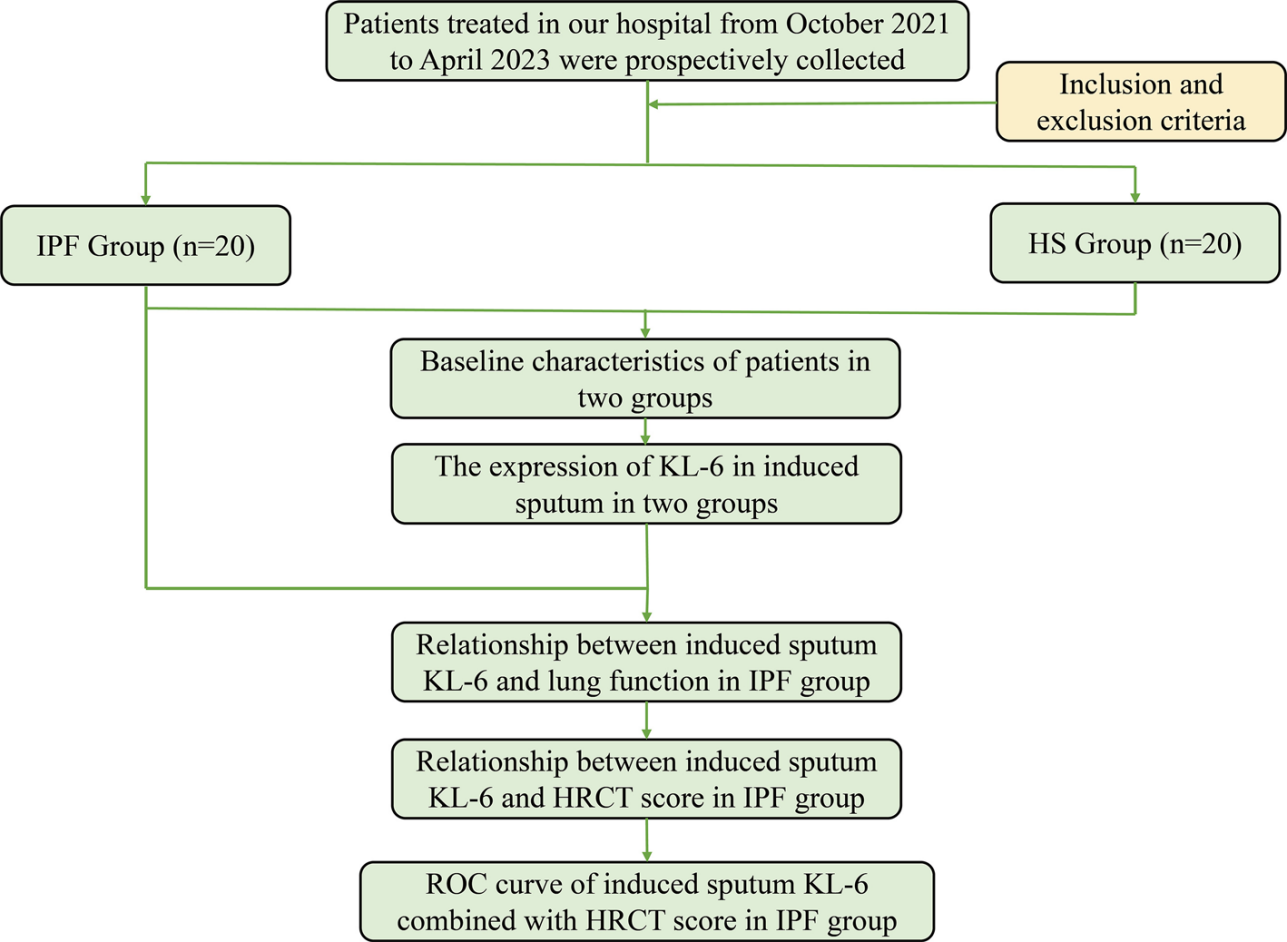
**Flowchart of the study population.** Abbreviations: IPF: Idiopathic pulmonary fibrosis; HS: Healthy subjects; KL-6: Krebs von den Lungen-6; HRCT: High-resolution computed tomography; ROC: Receiver operating characteristic.

The exclusion criteria for this study included: patients with IPF combined with chronic obstructive pulmonary disease (COPD), emphysema, pulmonary infections, or acute lung injury; patients with secondary ILDs such as connective tissue disease-associated ILD (CTD-ILD), those with occupational or environmental exposures, chronic hypersensitivity pneumonitis, and similar conditions; as well as pregnant or lactating women. Healthy subjects were recruited from the health examination population at our hospital’s physical examination center during the same period as the IPF patients. Inclusion criteria for healthy subjects included age-matching with the IPF groups, absence of lung disease or other major systemic illnesses, no respiratory symptoms in the past week, and no history of medication use. The study protocol is illustrated in Figure 1. This research was approved by the Ethics Committee of our hospital (2020016). Prior to participation, each patient provided written informed consent.

In the diagnostic process for disease identification, we facilitated multidisciplinary discussions involving the director of rheumatology and immunology, the attending physician from the imaging department, and respiratory specialists. A comprehensive analysis of the patient’s clinical symptoms, physical examination findings, rheumatoid immune antibody results, and HRCT imaging was conducted. For patients exhibiting usual interstitial pneumonia (UIP) or potential UIP patterns, a clinical diagnosis of IPF was established after excluding rheumatic immune diseases.

Sample size estimation was conducted utilizing G*Power software, employing an independent samples *t*-test model. The analysis incorporated the following parameters: an effect size (Cohen’s d) of 0.9, a significance level (α) of 0.05, and a statistical power of 0.80. The results indicated that a minimum of 20 participants per group, resulting in a total sample size of 40, would be necessary for the study.

### Research program

#### Sample collection and KL-6 testing

Patients inhaled 400 µg of salbutamol prior to the collection of induced sputum to facilitate bronchial dilation. Subsequently, they inhaled a hypertonic solution (5% NaCl) to induce sputum production. Throughout the induction process, forced expiratory volume in one second (FEV1) was monitored. If FEV1 decreased by more than 20% from baseline, the procedure was halted to ensure patient safety. Following sputum collection in a plastic container, the sample was weighed and vortexed for 30 s after adding three times its volume of phosphate-buffered saline (PBS). The mixture was then centrifuged at 800 g for 10 min at 4 ^∘^C. After separating the supernatant, samples were stored at −80 ^∘^C [17, 18]. Notably, dithiothreitol was not utilized during sputum processing; instead, viscosity was effectively reduced through dilution with PBS and vortex mixing, ensuring sample suitability. Samples containing less than 20% squamous cells were deemed eligible for the study [19].

We assessed KL-6 levels in sputum samples using the KL-6 enzyme immunoassay kit (Bioswamp, China). Initially, we performed a serial dilution to create a concentration gradient of the standard substance, which was diluted sequentially to six concentrations: 1800, 900, 450, 225, 112.5, and 0 U/mL. Each concentration was tested in duplicate on an ELISA plate, allowing for the establishment of a calibration curve.

Subsequently, we added 40 µL of the sample and 10 µL of biotinylated anti-KL-6 antibody to the test wells. In the blank control wells, the same procedure was followed, omitting the sample and antibody. We then added 50 µL of ELISA reagent to all reaction wells and incubated the plate in a 37 ^∘^C water bath for 30 min. After discarding the solution, the plate was washed five times with a 30-fold diluted washing solution.

Next, we added 50 µL of Developer A followed by Developer B to each well, mixing thoroughly and incubating in the dark at 37 ^∘^C for 10 min. The reaction was terminated by adding 50 µL of termination solution, resulting in a color change from blue to yellow. The optical density (OD) of the blank wells was set to zero, and we measured the OD values of each well at a wavelength of 450 nm within 15 min. The concentration of KL-6 in the samples was calculated based on the standard curve.

Precision was assessed using low-level quality control (CV = 3.95%) and high-level quality control (CV = 3.12%), with an overall intra-batch CV ≤ 8.0% and inter-batch CV ≤ 15.0%, demonstrating the method’s good repeatability and reproducibility.

#### Tests for pulmonary function

We implemented pulmonary function tests in accordance with the guidelines provided by the ATS [20]. Employing the Viasys Master Screen Vegeta/DFF (Viasys Healthcare GmbH, Höchberg, Germany), we performed standard spirometry for all patients, measuring key parameters such as FVC, FEV1, and the FEV1/FVC ratio. Additionally, we assessed the DL_CO_ to evaluate lung diffusion function, with all results expressed as percentages of actual and predicted values. FEV1% predicted values above 70% were classified as normal, 60%–70% as mild impairment, 50%–60% as moderate impairment, and below 50% as severe impairment. DL_CO_% predicted values above 80% were deemed normal, 60%–80% as mild impairment, 40%–60% as moderate impairment, and below 40% as severe impairment.

#### HRCT score

All patients underwent HRCT of the lungs using a 64-row, 128-slice multidetector computed tomography (MDCT) system (Siemens Somatom Definition, Siemens, Germany). The CT images and imaging parameters were standardized across all patients, featuring a tube voltage of 120 kVp, scan collimation of 128×0.6 mm or 256×0.6 mm, tube current modulation, a reconstructed slice thickness of 1.5 mm, and a gantry rotation speed of 0.5 s per rotation. The scan range included the lung apices down to the lower half of the diaphragm.

HRCT images of patients with IPF were evaluated by two radiologists, each possessing over two years of experience. The mean score from these evaluations was considered the final result. Fibrosis was assessed using a six-level scoring system, with the lungs divided into six regions: the area above the aortic arch, the section below the aortic arch to the right pulmonary vein, and the region below the right pulmonary vein, encompassing the upper, middle, and lower lung zones [21]. Utilizing the international HRCT grading method [22, 23], the score for interstitial abnormalities in each lung zone ranged from 0 to 5, contributing to a total possible score of 30. The specific scoring criteria were as follows: score 0 (no significant interstitial changes observed); score 1 (abnormal lobular structures, such as subpleural arc shadows and vertical line shadows); score 2 (abnormal interstitial shadows around bronchovascular bundles, thickening of these bundles, lobular contour deformation, and lobular septum changes); score 3 (abnormal lobular morphology with interstitial shadow abnormalities around bronchovascular bundles and honeycomb shadows measuring <10 mm); score 4 (honeycomb shadows expanded to 10–30 mm); and score 5 (abnormal interstitial shadows surrounding small bronchovascular bundles, with honeycomb shadows >30 mm and unclear or absent lung texture). The cumulative HRCT scores reflected the fibrosis scores for each lung region, allowing for a quantitative analysis of the degree of pulmonary fibrosis in patients based on HRCT imaging.

### Ethical statement

The study was approved by the ethics committee of the First People’s Hospital of Fuyang District (2020016). The methods were carried out in accordance with the approved guidelines.

**Table 1:** Baseline clinical characteristics of patients with idiopathic pulmonary fibrosis (IPF) and healthy subjects (HS)

	**IPF (*n* ═ 20)**	**HS (*n* ═ 20)**	***P* value**
Age (years)	73.00 (67.25, 77.00)	68.50 (63.50, 73.75)	0.184
Sex			0.311
Male	15 (75.0%)	12 (60.0%)	
Female	5 (25.0%)	8 (40.0%)	
BMI (kg/m^2^)	24.77±5.15	23.71±5.43	0.530
Smoke			0.525
Yes	12 (60.0%)	10 (50.0%)	
No	8 (40.0%)	10 (50.0%)	
Complication			1.000
Hypertension	6 (30.00)	7 (35.00)	
Diabetes	9 (45.00)	8 (40.00)	
Coronary heart disease	6 (30.00)	5 (25.00)	
CRP	3.63 (0.46, 9.04)	4.65 (1.66, 8.95)	0.565
SpO_2_	92.00 (90.25, 93.75)	97.00 (95.25, 98.75)	<0.001
Pulmonary function parameters			
FEV1	1.45 (1.31, 1.67)	/	
FVC	1.79 (1.59, 2.03)	/	
FEV1/FVC	0.82 (0.79, 0.84)	/	
FEV1%pred	59.12 (55.72, 63.41)	/	
FVC%pred	68.58 (65.28, 70.48)	/	
DL_CO_	4.21 (3.63, 4.64)	/	
DL_CO_%pred	63.50 (55.00, 71.00)	/	
DL_CO_/VA%	61.50 (56.00, 68.75)	/	
GAP stage			
I	7 (35.00)	/	
II	11 (55.00)	/	
III	2 (10.00)	/	
HRCT score	9.00 (8.00, 11.00)	0.50 (0.50, 1.38)	<0.001

Abbreviations: IPF: Idiopathic pulmonary fibrosis; HS: Healthy subjects; BMI: Body mass index; CRP: C-reactive protein; SpO: Peripheral capillary oxygen saturation; FEV1: Forced expiratory volume in one second; FVC: Forced vital capacity; FEV1/FVC: Ratio of forced expiratory volume in one second to forced vital capacity; DL_CO_: Diffusing capacity of the lung for carbon monoxide; DL_CO_/VA%: Diffusing capacity of the lung for carbon monoxide per unit of alveolar volume; GAP: Gender–age–physiology; HRCT: High-resolution computed tomography.

### Statistical analysis

Data were analyzed using IBM SPSS Statistics version 26.0 (IBM Corporation, USA). Initially, all measurement data were assessed for normality through the Shapiro-Wilk test. Quantitative data exhibiting a normal distribution were presented as mean ± standard deviation (SD). The independent samples *t*-test was employed for comparisons between two groups. For quantitative data that did not follow a normal distribution, results were expressed as median (quartiles) [M (P25, P75)]. The non-parametric Mann–Whitney *U* test was utilized for group comparisons, while ANOVA facilitated multi-group comparisons. Categorical data were reported as *N* (%), with group comparisons conducted using the chi-square (χ^2^) test. All statistical tests were two-tailed, with a significance level set at *P* < 0.05.

Given that the HRCT score was treated as an ordered categorical variable, Spearman correlation analysis was conducted to evaluate the relationship between induced sputum KL-6 levels, lung function, and HRCT scores. Receiver operating characteristic (ROC) curve analysis was employed to assess the diagnostic efficacy of induced sputum KL-6 and the combined HRCT score for IPF. Diagnostic accuracy was quantified by calculating the area under the curve (AUC), with the optimal ROC threshold determined by the maximum Youden index. To mitigate the risk of model overfitting and bias, internal validation of the ROC curve was performed using the bootstrap method with 1000 repeated samples, yielding robust estimates of AUC and its 95% confidence interval (CI), thereby enhancing the reliability of the diagnostic efficacy evaluation.

## Results

### Baseline characteristics of patients

The clinical characteristics of patients in the two groups were analyzed (Table 1). In the IPF group, the median age was 73 years, with 15 males (75.0%) and 5 females (25.0%). The mean body mass index (BMI) was 24.77 ± 5.15 kg/m^2^, and 12 patients reported a history of smoking. Additionally, 14 patients presented with comorbidities, including 6 cases of hypertension, 9 cases of diabetes, and 6 cases of coronary heart disease. The median oxygen saturation (SpO_2_) level in the IPF group was significantly lower than that of the healthy group (92.00 vs 97.00, *P* < 0.001). Furthermore, a significant difference in HRCT scores was observed between the two groups (9.00 vs 0.5, *P* < 0.001). No significant differences were found in age, gender, BMI, smoking history, comorbidities, or C-reactive protein (CRP) levels between the two groups (*P* > 0.05).

### Induced sputum KL-6 is greatly elevated in IPF patients

**Figure 2.**
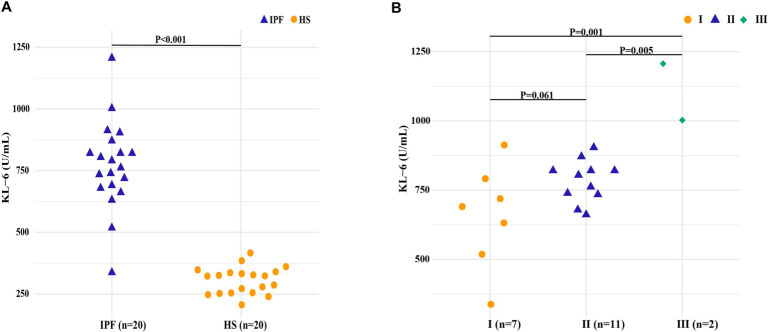
**Induced sputum KL-6 levels in IPF patients and healthy subjects (HS).** (A) Comparison of induced sputum KL-6 levels between the two groups; (B) Induced sputum KL-6 levels across different GAP stages in the IPF group. Note: Data are presented as scatter plots with individual values. III P75 could not be computed due to small sample size (*n* ═ 2). Abbreviations: IPF: Idiopathic pulmonary fibrosis; HS: Healthy subjects; KL-6: Krebs von den Lungen-6.

The IPF group exhibited significantly elevated KL-6 levels in induced sputum at 776.29 (IQR: 681.98, 858.57) U/mL, compared to the healthy subjects (HS) group, which had levels of 322.21 (IQR: 253.67, 338.64) U/mL (*P* < 0.001) (Figure 2A). Stratification of IPF patients by the GAP index severity stages revealed notable differences in KL-6 levels across the stages: Stage I exhibited levels of 690.44 (IQR: 518.20, 790.65) U/mL, Stage II showed 804.39 (IQR: 734.42, 820.83) U/mL, and Stage III reached 1104.60 (IQR: 1104.60, NA) U/mL, with statistically significant differences observed among the groups (Figure 2B, *P* < 0.05).

### Relationship between induced sputum KL-6 and lung function and HRCT score in the IPF group

**Figure 3.**
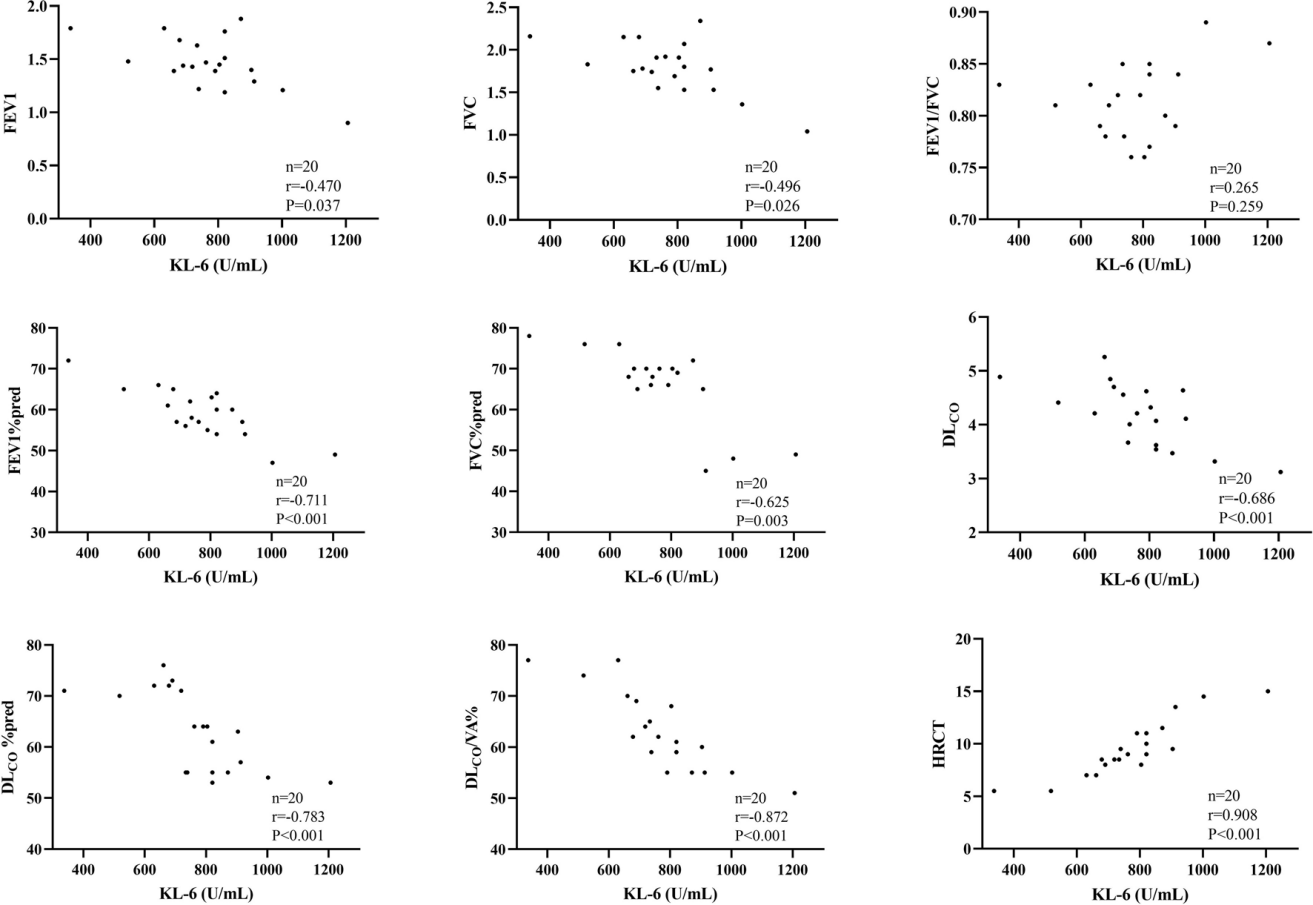
**Correlations between induced sputum KL-6 levels, lung function parameters, and HRCT scores in patients with IPF.** Scatter plots illustrate significant negative correlations between KL-6 and FEV1, FVC, FEV1%pred, FVC%pred, DL_CO_%, DL_CO_%pred, and DL_CO_/VA%, as well as a strong positive correlation with HRCT scores. Corresponding correlation coefficients (r) and *P* values are shown within each panel. Abbreviations: KL-6: Krebs von den Lungen-6; FEV1: Forced expiratory volume in one second; FVC: Forced vital capacity; FEV1/FVC: Ratio of forced expiratory volume in one second to forced vital capacity; DL_CO_: Diffusing capacity of the lung for carbon monoxide; DL_CO_/VA%: Diffusing capacity of the lung for carbon monoxide per unit of alveolar volume; HRCT: High-resolution computed tomography.

In the IPF group, levels of induced sputum KL-6 exhibited a significant negative correlation with various lung function parameters, including FEV1 (*r* ═ –0.470, *P* ═ 0.037), FVC (*r* ═ –0.496, *P* ═ 0.026), FEV1% predicted (*r* ═ –0.711, *P* < 0.001), FVC% predicted (*r* ═ –0.625, *P* ═ 0.003), DL_CO_% (*r* ═ –0.686, *P* < 0.001), DL_CO_% predicted (*r* ═ –0.783, *P* < 0.001), and DL_CO_/VA% (*r* ═ –0.872, *P* < 0.001). These findings suggest a strong association between elevated induced sputum KL-6 levels and decreased lung function. Conversely, a significant positive correlation was observed between induced sputum KL-6 levels and the HRCT score (*r* ═ 0.908, *P* < 0.001) (Figure 3, Table S1).

### Analysis of induced sputum KL-6 and the extent of lung fibrosis on HRCT in the IPF group

**Figure 4.**
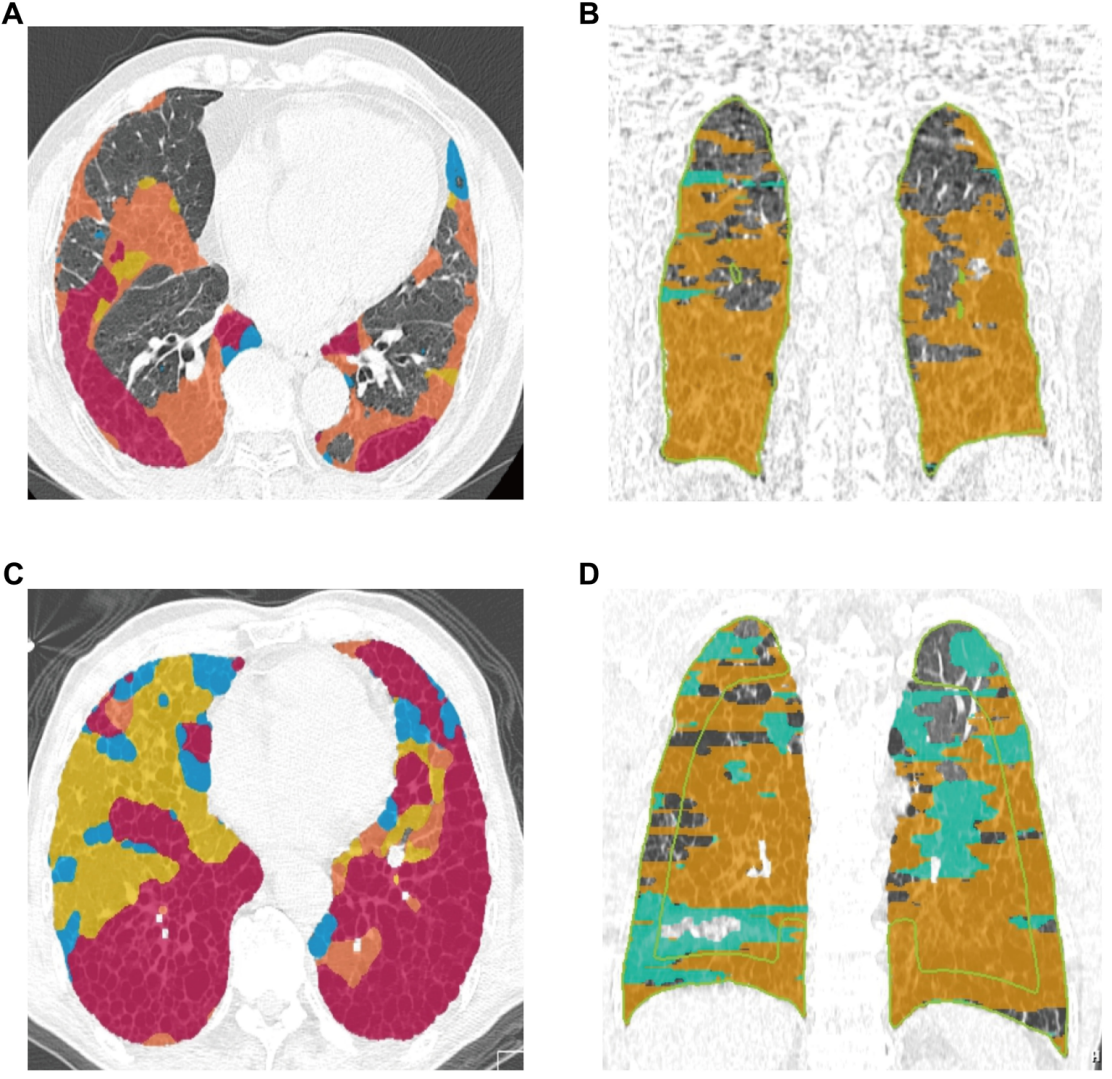
**HRCT assessment of lung fibrosis extent and corresponding induced sputum KL-6 levels in two IPF patients**. (A and B) Patient 1: HRCT revealed a lung fibrosis extent of 15.9%, with an induced sputum KL-6 level of 820.83 U/mL. Fibrotic areas are marked in color. (C and D) Patient 2: HRCT demonstrated a greater fibrosis extent of 31.3%, with a KL-6 level of 1002.63 U/mL, showing a consistent upward trend in KL-6 with increased fibrosis. Abbreviations: HRCT: High-resolution computed tomography; KL-6: Krebs von den Lungen-6; IPF: Idiopathic pulmonary fibrosis.

The lung fibrosis status of Patient 1 was assessed using HRCT, which revealed an extent of lung fibrosis at 15.9% and a serum KL-6 level of 820.83 U/mL (Figure 4A and 4B). In contrast, the HRCT images of Patient 2 indicated a total lung fibrosis extent of 31.3% along with a KL-6 level of 1002.63 U/mL (Figure 4C and 4D).

### ROC curves of induced sputum KL-6 combined with HRCT score

**Figure 5.**
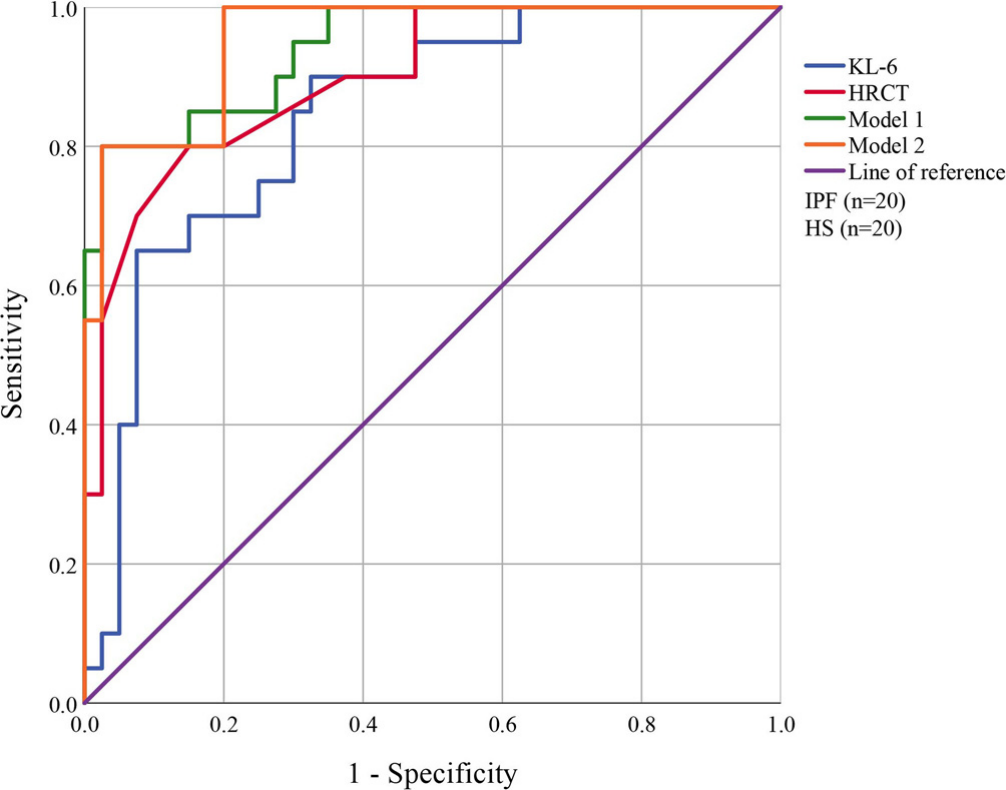
**ROC curves of induced sputum KL-6 and HRCT score in the diagnosis of IPF.** The diagnostic performance of induced sputum KL-6, HRCT score, and their combinations is shown. At a KL-6 threshold of 623.78 U/mL, sensitivity was 90.0%, specificity 67.5%, and AUC 0.844. An HRCT score of 7.75 yielded 80.0% sensitivity, 85.0% specificity, and an AUC of 0.899. Model 1 (KL-6 combined with HRCT) achieved 80.0% sensitivity, 97.5% specificity, and an AUC of 0.936 (95% CI: 0.914–0.944). After adjustment for confounding factors (age and smoking status), Model 2 showed 80.0% sensitivity, 97.5% specificity, and an AUC of 0.940 (95% CI: 0.874–0.956). Abbreviations: ROC: Receiver operating characteristic; IPF: Idiopathic pulmonary fibrosis; HS: Healthy subjects; KL-6: Krebs von den Lungen-6; HRCT: High-resolution computed tomography.

The ROC curves indicated that a KL-6 level of 623.78 U/mL in induced sputum achieved a sensitivity of 90.0% and a specificity of 67.5% for identifying the presence of IPF, with an AUC of 0.844 (Figure 5). In comparison, an HRCT score of 7.75 yielded a sensitivity of 80.0%, a specificity of 85.0%, and an AUC of 0.899 for IPF development. Conversely, Model 1 exhibited a specificity of 97.5% and a sensitivity of 80.0%, producing an AUC of 0.936 (95% CI: 0.914, 0.944) when combining both diagnostic indicators. Furthermore, we adjusted for confounding factors, including age and smoking status, in the joint diagnosis. Model 2 demonstrated a sensitivity of 80.0%, a specificity of 97.5%, and an AUC of 0.940 (95% CI: 0.874, 0.956) (Table 2).

**Table 2:** Diagnostic performance of induced sputum KL-6, HRCT score, and combined models for identifying IPF

	**AUC (95% CI)**	**Sensitivity**	**Specificity**
KL-6	0.844 (95%CI: 0.741–0.946)	0.900	0.675
HRCT	0.899 (95%CI: 0.818–0.980)	0.800	0.850
Model 1	0.936 (95%CI: 0.914, 0.944)	0.800	0.975
Model 2	0.940 (95%CI: 0.874, 0.956)	0.800	0.975

Note: Model 1: KL-6+HRCT, no adjusted; Model 2: Model 1 + adjusted age and smoke. Abbreviations: AUC: Area under the curve; CI: Confidence interval; KL-6: Krebs von den Lungen-6; HRCT: High-resolution computed tomography.

## Discussion

The project aimed to evaluate the potential role of KL-6 in induced sputum for the diagnosis and assessment of IPF and to investigate its association with pulmonary function parameters and HRCT scores. Comparative analysis between the IPF and HS groups revealed significantly elevated levels of KL-6 in induced sputum from IPF patients (*P* < 0.001), with increasing concentrations correlating with disease severity. Furthermore, KL-6 levels in induced sputum exhibited a significant negative correlation with pulmonary function parameters, including FEV1, FVC, FEV1 percentage predicted (FEV1%pred), FVC percentage predicted (FVC%pred), DL_CO_, DL_CO_ percentage predicted (DLCO%pred), and DLCO adjusted for alveolar volume (DL_CO_/VA%) (*P* < 0.05). In addition, there was a significant positive correlation between KL-6 levels and HRCT scores (*P* < 0.001). The diagnostic efficacy of KL-6 in conjunction with HRCT scores was notably high, with an AUC of 0.936 (95% CI: 0.914, 0.944), and sensitivity and specificity values of 80.0% and 97.5%, respectively.

Patients with IPF exhibit elevated levels of KL-6 due to significant damage and fibrosis of alveolar epithelial cells, which serves as a crucial basis for early diagnosis and disease monitoring of IPF. In this study, KL-6 levels in induced sputum were found to be higher than those in the IPF group, corroborating previous research that identified KL-6 as a marker for ILD [11, 24, 25]. Elevated KL-6 levels are indicative of both the severity and progression of ILD [26]. A meta-analysis indicated that the KL-6 level in patients with severe ILD averages 703.41 U/mL, which is significantly higher than that in patients with mild disease. Furthermore, KL-6 levels in progressive ILD patients average 325.98 U/mL, markedly exceeding those in non-progressive patients [27]. Additionally, KL-6 levels have prognostic implications. Yokoyama et al. [26] demonstrated that IPF patients with low KL-6 levels often survive for over 36 months, while those with high KL-6 levels typically have a survival duration of only 18 months. This suggests that serum KL-6 assessment can effectively predict the survival rates of IPF patients. We hypothesize potential reasons for the elevation of KL-6 levels.

First, the pathological damage associated with IPF and ILD is diverse, with the most common forms being pulmonary vascular epithelial damage and diffuse alveolar epithelial cell damage. These injuries can induce chronic fibrosis [28]. Secondly, KL-6 is an immunoglobulin glycoprotein primarily regulated by the *MUC1* gene, exhibiting high expression on proliferating alveolar epithelial cells type II (AECII). When AECII are damaged or regenerated, KL-6 on the cell membrane is readily sheared and released into the alveolar cavity and airway secretions. If the alveolar-capillary barrier is compromised, KL-6 can enter the bloodstream [29, 30]. Thirdly, in severe cases of IPF-ILD, an extensive inflammatory response and exudation from degenerative AECII not only lead to an expansion of lung parenchyma visible on HRCT but also stimulate the release of KL-6 from additional AECII [31].

Induced sputum KL-6 levels exhibited a significant inverse correlation with lung function parameters, including FEV1, FVC, FEV1%pred, FVC%pred, DL_CO_%, DL_CO_%pred, and DL_CO_/VA%. This finding indicates that KL-6 not only reflects the extent of lung inflammation and damage but also influences the actual lung function of patients. Thus, KL-6 levels may serve as a crucial indicator for assessing lung function impairment in patients with IPF. Sokai et al. [32] noted that variations in serum KL-6 levels over a six-month period were significantly associated with changes in FVC (*r ═* –0.38, *P* < 0.01) and DL_CO_% (*r ═ –*0.33, *P* ═ 0.01) in IPF patients. Additionally, a case-control study demonstrated that elevated serum KL-6 levels were negatively correlated with FVC (*r* ═ –0.93) and FEV1 (*r* ═ –0.91) [33]. Collectively, these findings support the potential of KL-6 as a valuable biomarker for evaluating lung function.

Induced sputum levels of KL-6 were significantly positively correlated with HRCT scores (*P* < 0.001). This finding aligns with the results of Wang et al. [34], which indicated that KL-6 levels reflect the degree of fibrosis observed in imaging studies, and that measuring KL-6 in induced sputum simplifies the complexities associated with disease monitoring.

Our study included two patients with typicalUIP imaging. The HRCT image of patient 1 revealed a pulmonary fibrosis extent of 15.9%, correlating with KL-6 levels of 820.83 U/mL. In contrast, patient 2 exhibited a pulmonary fibrosis extent of 31.3%, with KL-6 levels rising to 1002.63 U/mL. This data indicates a consistent upward trend in KL-6 levels corresponding to the degree of fibrosis assessed by HRCT.

We hypothesize that the elevation of KL-6, a high molecular weight glycoprotein secreted by alveolar type II cells, reflects damage and regeneration of alveolar epithelial cells. Patients with greater levels of fibrosis, such as patient 2, often experience more extensive alveolar epithelial damage and remodeling, leading to increased KL-6 secretion and significantly elevated detection levels. These cases suggest that detecting KL-6 in induced sputum not only reflects local pathological changes in the lower respiratory tract but may also be closely associated with the extent of fibrosis assessed through imaging.

The diagnostic efficacy of KL-6 in ILD has been validated, with ROC curves indicating a sensitivity of 76.36% and specificity of 91.07% [34]. This suggests that induced sputum KL-6 demonstrates higher sensitivity, reinforcing its potential as a reliable biomarker for assessing lung fibrosis. Additionally, Ohshimo et al. [24] established that serum KL-6 levels exceeding 1300 U/mL can serve as a predictive biomarker for acute exacerbation of IPF, exhibiting a sensitivity of 92% and specificity of 61%. In our ROC analysis, induced sputum KL-6 levels were measured at 623.78 U/mL, yielding a sensitivity of 90.0% and specificity of 67.5%, with an AUC of 0.844. When combined with HRCT scoring analysis, specificity increased further, resulting in an AUC of 0.943, thereby significantly enhancing diagnostic accuracy. Although the integration of KL-6 and HRCT scoring markedly improved the AUC, its clinical value extends beyond statistical enhancement; it also highlights the complementary benefits of combining biological markers with imaging findings. Furthermore, after adjusting for variables such as age and smoking status, the results remained supportive. This indicates that the combined application of induced sputum KL-6 and HRCT scoring holds promise for more effective identification of IPF patients, enhancing both the sensitivity and specificity of diagnosis while providing comprehensive support for clinical decision-making.

This study has several limitations. First, the sample size is relatively small. Although we employed non-parametric analysis methods and implemented strict inclusion and exclusion criteria to minimize data bias, individual differences may still influence the results. Future research should utilize multivariate regression models with multi-center, large-scale cohorts to better control for confounding factors and validate the clinical predictive value identified in this study. Additionally, external independent cohort validation will be conducted to assess the model’s broader applicability.

Second, the control group consisted solely of healthy individuals, which limits our ability to comprehensively evaluate the differential diagnostic capability of KL-6 in complex clinical scenarios involving other ILDs or chronic respiratory diseases. Future studies will incorporate disease control groups to systematically assess the sensitivity and specificity of induced sputum KL-6 across various conditions, thereby elucidating its practical application value in auxiliary diagnosis.

Third, this study did not collect serum KL-6 data concurrently, preventing a direct comparison of the diagnostic and evaluative advantages of serum vs induced sputum KL-6. Future research will conduct comparative studies to clarify their complementarity and clinical relevance.

Fourth, although all patients underwent chest CT examinations at enrollment to rule out pulmonary infection, and CRP data were provided to further support the absence of active infection during sampling, procalcitonin data were missing in over 30% of cases and were not analyzed. Consequently, this study cannot entirely exclude the potential impact of inflammation or infection on KL-6 levels, necessitating caution in result interpretation. Future investigations will integrate KL-6 testing with clinical data (including CRP, procalcitonin, symptoms, and imaging results) to clarify the influence of inflammatory confounding factors.

Finally, the absence of follow-up data limited our ability to systematically collect longitudinal dynamic changes in KL-6, restricting in-depth analysis of its value in monitoring disease progression and prognostic evaluation. Future prospective long-term follow-up studies will be essential to comprehensively assess the diagnostic and monitoring potential of KL-6.

## Conclusion

This study investigates the potential application of induced sputum KL-6 as a diagnostic and evaluative tool for IPF. The findings reveal that KL-6 levels in induced sputum are significantly elevated in patients with IPF compared to healthy subjects. Furthermore, sputum KL-6 levels correlate closely with lung function parameters and HRCT scores. The integration of KL-6 measurements with HRCT scoring enhances the diagnostic accuracy for IPF, offering valuable insights for clinical diagnosis and management. This research suggests that induced sputum KL-6 may serve as a promising biomarker for IPF. Nonetheless, further validation in larger sample sizes and multicenter cohorts is necessary, along with an exploration of its clinical utility to optimize the diagnosis and management of IPF.

## Supplemental data

**Table S1:** Relationship between induced sputum KL-6 and lung function and HRCT score

	***r***	***P* value**
FEV1	–0.470	0.037
FVC	–0.496	0.026
FEV1/FVC	0.265	0.259
FEV1%pred	–0.711	<0.001
FVC%pred	–0.625	0.003
DL_CO_	–0.686	<0.001
DL_CO_%pred	–0.783	<0.001
DL_CO_/VA%	–0.872	<0.001
HRCT	0.908	<0.001

Abbreviations: FEV1: Forced expiratory volume in one second; FVC: Forced vital capacity; FEV1/FVC: Ratio of forced expiratory volume in one second to forced vital capacity; DL_CO_: Diffusing capacity of the lung for carbon monoxide; DL_CO_/VA%: Diffusing capacity of the lung for carbon monoxide per unit of alveolar volume; HRCT: High-resolution computed tomography.

## Data Availability

All data generated or analyzed during this study are included in this article.
